# Streptococcus pneumoniae Spinal Epidural Abscess in an Immunocompetent Patient

**DOI:** 10.7759/cureus.68710

**Published:** 2024-09-05

**Authors:** Salena Sinnasone, Michelle Blyth

**Affiliations:** 1 School of Medicine, Louisiana State University Health Sciences Center, New Orleans, USA; 2 Infectious Disease, Tulane University School of Medicine, New Orleans, USA

**Keywords:** epidural abscess, immunocompetent patients, invasive pneumococcal infection, rare manifestations of infections, streptococcus pneumonae

## Abstract

Epidural abscesses are a rare diagnosis in immunocompetent patients. The most common risk factors linked with spinal epidural abscesses are intravenous drug use, diabetes mellitus, hepatitis, and iatrogenic factors like prior surgery and catheter usage. The thoracic and lumbar spine are the most common sites of these abscesses. Clinical presentation can include back pain, fever, and neurologic deterioration, with back pain occurring in almost two-thirds of patients. *Staphylococcus aureus* is the most common causative pathogen.

We present a 50 male with no significant past medical or family history who presented with progressive back pain for greater than one week, chills and malaise. Cervical and lumbar spinal CT scans identified epidural abscesses at C6/7 and L5/S1. Blood cultures and surgical cultures from washout of the epidural space grew *Streptococcus pneumoniae*. The patient was treated successfully with a prolonged course of cefazolin for six weeks.

*S. pneumoniae* is a rare cause of epidural abscesses, especially in patients with no known risk factors for invasive disease. This case demonstrates that invasive pneumococcal disease should remain on the differential diagnosis even in immunocompetent patients.

## Introduction

*Streptococcus pneumoniae* is a gram-positive, lancet-shaped, opportunistic bacteria [1,2]. It is one of the most common causes of community-acquired pneumonia worldwide, with seasonal increases in winter and early spring. It can also cause invasive pneumococcal disease when *S. pneumoniae* invades otherwise sterile sites causing bacteremia, meningitis, osteomyelitis, and other invasive diseases [3]. These diseases are most common, and most severe, in patients older than 65 years of age, children younger than 2, and individuals with risk factors such as smoking, alcohol abuse, Chronic Obstructive Pulmonary Disease (COPD), or asplenia [1]. *S. pneumoniae* is the cause of over 300,000 deaths of children under the age of 5 worldwide each year, and in 2020, invasive disease accounted for over 2,000 deaths in the United States [3,4]. 

Risk factors for *S. pneumoniae* pneumonia as well as invasive disease include alcoholism, cerebrospinal fluid leak, cigarette smoking, cochlear im1plant, immunocompromise, diabetes, functional or anatomic asplenia (including sickle cell disease), and chronic heart, lung, liver, or renal disease [3]. Certain ethnic and racial groups have an increased risk of the disease including Alaskan Natives, African Americans, and certain American Indian people [3]. Increased levels of chronic stress due to racism and poverty within communities contribute to decreased immune function and are a possible factor for increased disease incidence [5]. Pneumococcal vaccination rates are also lower among Black and Hispanic adults [5].

Epidural abscesses occur at a rate of 0.2-2.8 cases per 10,000 per year with presentation in immunocompetent patients is even rarer [6,7]. The classic triad of symptoms in patients with spinal epidural abscesses includes back pain, fever, and neurologic deficits [8,9]. The most common organism isolated in these abscesses is Staphylococcus aureus [6,7].

Pneumococcal spinal lesions, especially in immunocompetent patients without risk factors, are rarely seen [10]. A literature review of pneumococcal spinal infections from 1906 to 2012 revealed that localized back pain in adults was the most universal symptom described by patients [11]. Sixty-two percent of patients reported a fever and only 50% of those patients with pneumococcal spinal infections presented with neurologic deficits [11].

## Case presentation

We present a 50-year-old male with no known past medical history who presented to the hospital with greater than one week of progressive back pain associated with chills and malaise. He started having midline lower back pain that began acutely and spontaneously several weeks ago without any trauma or other complaints. The pain then progressed to both of his shoulders, wrists, and chest. The patient detailed having multiple emergency room visits within the past week due to back pain as well as some chest discomfort. These visits included blood work but no back imaging. At his last visit, blood cultures were drawn. He explained that he was told no acute process was found and he was discharged with anti-inflammatories that improved his pain but did not resolve it.

On a review of symptoms, he denied any shortness of breath, cough, sinus pain, or drainage. He also denied headaches, photophobia, or neck stiffness. He denied difficulty ambulating, urinary or bowel incontinence, tingling, or numbness. He endorsed daily marijuana use (inhalation) and denied all other drug use including alcohol, tobacco, and intravenous drug use. He also denied a history of sickle cell anemia, asplenia, prior infections, steroid use, head or other trauma, antibiotic use, or immunocompromising conditions. He denied prior hospitalizations or other disease history. His family history was reviewed which was noncontributory with no history of immunocompromise or frequent infections in his family which included his son and parents.

On physical exam, the patient was afebrile and hemodynamically stable with no distress. The pulmonary exam was benign without crackles, rales, or decreased breath sounds. No abnormalities of the head, ears, dentition, nose, or mouth were found. He was found to have tenderness over his bilateral shoulders and left wrist. No tenderness, edema, fluctuance, or erythema was noted over his spine, wrist, or shoulders. All joints had with normal range of motion, including the spine and neck. No neck stiffness or pain on movement was found. The patient was well nourished but not overweight. No neurological deficits were present. Overall, no significant abnormalities were found.

**Figure 1:**
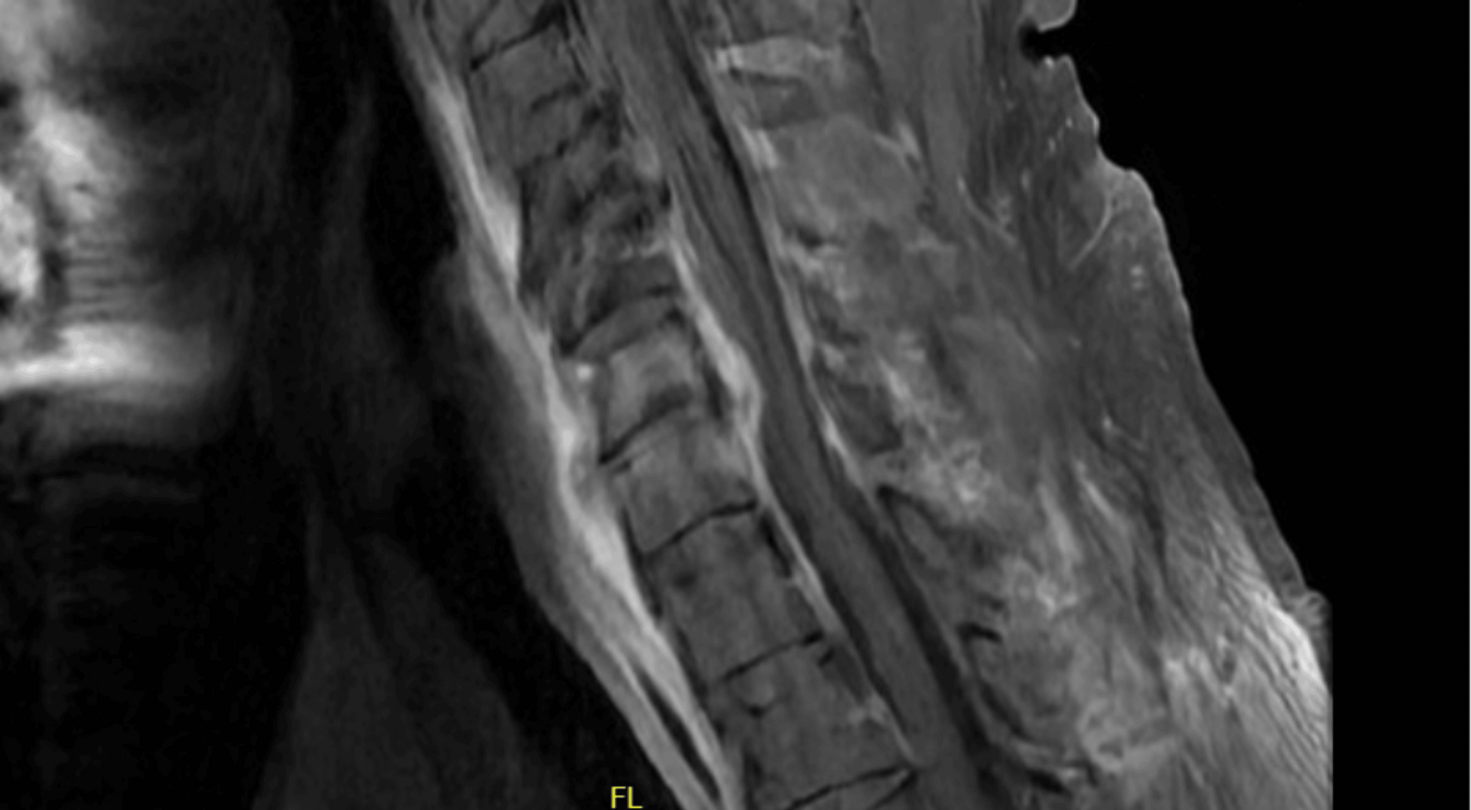
Cervical spinal CT revealing a developing epidural abscess with osteomyelitis at C6/7.

**Figure 2:**
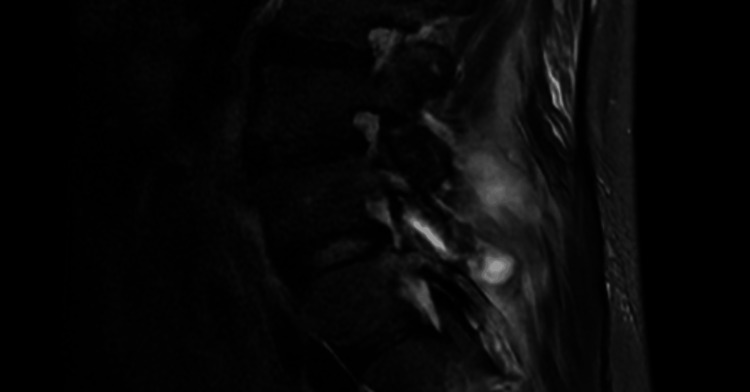
The lumbar spine CT is suspicious for a loculated epidural abscess at the L5 and S1 levels. There is a peripherally enhancing fluid collection in the left erector spinal muscle at the level of L5 and S1, which is suspicious for an abscess.

His complete blood count (CBC) revealed a white blood cell (WBC) count of 27 x 10^9^/L (4-10), and upon review of prior CBCs, it was noted that his WBC had been increasing since his first ED visit. Other notable laboratory results included a mildly elevated procalcitonin level of 0.57 ng/mL (reference range: 0-0.10), C-reactive protein (CRP) of 37.8 mg/dL (<1.0), erythrocyte sedimentation rate (ESR) of >150 mm/hour (0-20), and a quantitative D-dimer of 2,515 ng/mL (<500). Admission blood cultures gathered during his last emergency room visit were positive for penicillin-sensitive *S. pneumoniae*. Cervical and lumbar spine CTs showed an epidural abscess at C6/7 and L5/S1, as seen in Figure 1 (cervical spine CT) and Figure 2 (lumbar spine CT).

The patient was taken for a spinal washout, and cultures taken during surgery grew rare *S. pneumoniae*.

A transthoracic echocardiogram was performed to rule out endocarditis as the patient had multifocal spinal abscesses, despite *S.* *pneumoniae* being a rare cause of such. This showed no vegetation or valvular compromise. A CT chest revealed no septic emboli, consolidations, or other signs of pneumonia.

Blood immunoglobin enzyme-linked immunosorbent assay (ELISA) was obtained to rule out common variable immunodeficiency (CVID) and resulted in elevated IgG with normal ranges of IgM and IgA. An HIV-1/2 Ag/Ab Combo test was also obtained which was negative. White blood cell count differential showed increased neutrophils but no lymphopenia or other significant abnormalities. A1C was within normal levels as was creatinine. 

The patient was successfully treated with IV ceftriaxone 2 g daily for a total of six weeks based on a literature review of similar cases, ease of dosing, and narrow spectrum. Pneumococcal vaccination was recommended as an outpatient after recovery.

## Discussion

*S. pneumoniae* is a common commensal organism, especially in children. Approximately 27%-65% of children and less than 10% of adults have a commensal relationship with *S. pneumoniae *[7]. *S. pneumoniae *colonization of the upper respiratory tract occurs very early in childhood, beginning in the first few months of life [12]. Typically, individuals with a competent immune system can control *S. pneumoniae* without significant disease; however, immunocompromised individuals are susceptible to horizontal dissemination of *S. pneumoniae* into the lower airways, which can lead to invasive disease [12,13]. Symptomatic infection can present after the nasopharynx or oropharynx is colonized but usually remains asymptomatic unless the patient has predisposed risk factors or they are colonized with a particularly virulent strain [6]. 

Children under the age of 5, adults over 65 years of age, those with prior invasive pneumococcal disease, or individuals who have certain conditions or risk factors, including tobacco use, are recommended to be immunized against *S. pneumoniae *[8]. Rates of invasive pneumococcal disease have been decreasing after the introduction of the pneumococcal vaccines in both children and adults, though expectedly the proportion of cases caused by nonvaccine strains has increased [14,15]. In addition to decreasing cases, the introduction of pneumococcal conjugate vaccines has resulted in a decrease in antibiotic-resistant pneumococcal infections [15].

This case demonstrates the variability of invasive *S. pneumoniae* disease and the importance of considering this pathogen in presumed immunocompetent adult hosts, even in patients without respiratory signs or symptoms. Workup in these cases should include testing for immunocompromising conditions, including HIV and CVID, as was done in this case. After disease resolution, patients with a history of invasive *S. pneumoniae *should be offered vaccination to prevent recurrent invasive disease. While tobacco use is considered an indication for vaccination, currently marijuana inhalation is not [8]. Further investigation should be considered to determine the risks of invasive *S. pneumoniae *in marijuana users and if it should be an indication for vaccination. In addition, research investigating invasive pneumococcal disease about patient risk factors, as well as changing strain prevalence, may help us further understand these rare disease presentations.

## Conclusions

This is a rare presentation of invasive pneumococcal disease and spinal epidural abscess in an otherwise healthy middle-aged male with no risk factors outside of daily marijuana inhalation. Few similar cases have been described in the literature, with only one patient with no known risk factors who developed an *S. pneumoniae* epidural abscess. This patient also did not exhibit neurologic deficits in his presentation which has only been recognized in six other cases before this one.

This case highlights the importance of considering spinal epidural abscesses in patients with new onset back pain, even younger patients without immunocompromising conditions or risk factors. It also highlights that invasive pneumococcal disease can be subtle and unpredictable even in patients without known risk factors. Although vaccines have significantly decreased invasive *S. pneumoniae* disease, unusual cases can still be found.
